# The impact of dietary vegetable oil sources with or without vitamin E supplementation on the productive performance and egg yolk lipids of quail breeders

**DOI:** 10.5194/aab-69-665-2026

**Published:** 2026-11-12

**Authors:** Nahed S. H. Abo Egila, Waleed M. Dosoky, Mohamed H. Ahmed, Soliman M. Zahran, Ahmed Sh. Awad, Ayman S. Salah, Mohamed S. El-Kholy, Maha Abdelhaseib, Mohamed M. Habib, Mahmoud Alagawany, Mahmoud M. Azzam

**Affiliations:** 1 Department of Animal and Fish Production, Faculty of Agriculture (Saba Basha), Alexandria University, Alexandria, Egypt; 2 Animal Production Research Institute, Agriculture Research Center, Giza, Egypt; 3 Department of Nutrition and Clinical Nutrition, Faculty of Veterinary Medicine, New Valley University, El-Kharga, Egypt; 4 Department of Poultry, Faculty of Agriculture, Zagazig University, Zagazig, Egypt; 5 Animal Science Department, Purdue University, West Lafayette, IN 47906, USA; 6 Department of Animal Production, College of Food and Agricultural Sciences, King Saud University, Riyadh, Saudi Arabia

## Abstract

The goal of the present study was to discover the probable impact of sunflower oil (SUNO), linseed oil (LINO), and canola oil (CANO) with or without vitamin E treatments on the productive parameters and egg yolk lipid metabolism of quail eggs. In a completely randomized design, this trial was conducted as a 3 × 2 factorial trial with three sources of vegetable oils (SUNO 3 %, LINO 3 %, and CANO 3 %) and two types of vitamin E (0 and 250 mg kg^−1^ diet) in six treatments. A total of 192 female Japanese quails, 10 weeks of age, were randomly allocated to six dietary treatments, with 32 quails per treatment. Each treatment was further divided into four replicates of eight hens each. The experiment lasted for 10 weeks (10–20 weeks of age). The group of quails that received diets with canola oil at 15–20 weeks of age and linseed oil with vitamin E throughout the trial period (15–20 weeks of age) achieved the highest egg weight. The egg yolk malondialdehyde level was decreased at 15 and 20 weeks of age and after 15 and 30 d of storage in the refrigerator because of the addition of various vegetable oil sources in the diets. In summary, the productive criteria, lipid metabolism, and antioxidant properties of egg yolks in Japanese quail breeders (*Coturnix Japonica*) were all positively impacted by the combination of linseed oil, canola oil, and vitamin E during the laying period. Dietary supplementation with SUNO, LINO, and CANO improved the nutritive value of eggs by favorably modulating antioxidant stability and yolk metabolism lipids during refrigerated storage for 15 and 30 d, potentially participating in cardiovascular health and a more favorable dietary fatty acid profile.

## 1 Introduction

Dietary oils and other lipid sources are commonly incorporated into poultry diets because lipids provide approximately twice as much metabolizable energy (ME) as carbohydrates, making them an efficient source of dietary energy (Alhayaly et al., 2024). Numerous researchers observed no issues with the production efficacy of chickens when they were fed various dietary sources of n-3 polyunsaturated fatty acids (PUFAs) (Ebeid et al., 2008; Aziza et al., 2013; Saleh, 2013). In the poultry production sector, phytogenic feed supplements, which include their extracts and essential oils, are frequently used as alternatives to antibiotic growth enhancers (Saleh et al., 2019; Reda et al., 2024). Vegetable oils, including sunflower oil (SUNO), linseed oil (LINO), and canola oil (CANO), are valuable energy sources in laying-hen diets, providing highly essential fatty acids and digestible energy (Grobas et al., 2001). Their inclusion can improve feed utilization, egg yield, and egg weight while enhancing nutrient absorption and maintaining the composition of yolk fatty acids. Thus, appropriate vegetable oil supplementation may support productive performance, egg quality criteria, and sustainable production of laying hens (Gao et al., 2021).

The supplement of LINO in the feed considerably reduced the concentrations of cholesterol and triglycerides, as reported by Saleh et al. (2013) and Ibrahim and Abdul-Rahman (2024). Additionally, Promila et al. (2017) demonstrated that hens fed diets having 2.5 % linseed oil had significantly greater hen day egg production ( P < 0.05) than those fed a maize-based diet (control) and those of the other treatment method. In the same context, Celebi and Utlu (2006) reported that the egg production of chickens that were fed a diet with 4 % flaxseed oil was elevated ( P ≤ 0.05). The supplementation of linseed and linseed oil in the feed of poultry led to a 4 % rise in egg production (Augustyn et al., 2006). Conversely, Skřivan et al. (2008) additionally noted a reduction in egg production in chickens that were fed 3 % flaxseed oil. According to different studies, the link between the concentration of cholesterol and unsaturated lipid concentration in the feed must be taken into consideration when estimating the cholesterolgenic impacts of meals rather than only their cholesterol concentration (Hopkins, 1992; Alagawany et al., 2021). The liver is the most important site of cholesterol production in laying hens, where it is integrated into very low-density lipoprotein (VLDL) and vitellogenin (VTG) particles. These are subsequently discharged into the blood stream and absorbed by the developing oocytes through endocytosis. Subsequently, VLDL and VTG are included in the yolk, which accounts for a significant portion of the yolk cholesterol (Elkin, 2006). Linseed oil contains the highest amount of n-3 PUFAs, specifically alpha-linolenic acid (ALA) at roughly 45 % to 60 % of total fatty acids, followed by canola oil at around 8 % to 10 %, while sunflower oil contains negligible to very low amounts (Ghobadi et al., 2019; Alagawany et al., 2021; Marino et al., 2024). Therefore, poultry diets could be enriched by linseed oil (LO) and canola oil (CO), and the yolk will be enriched with ALA and docosahexaenoic acid-DHA (Alagawany et al., 2019; Abo Egila et al., 2023).

Vitamin E serves as the principal antioxidant agent in egg yolk lipids, efficiently halting the lipid peroxidation process by stabilizing the process of lipid peroxides via interaction (Domínguez et al., 2019). Nemati et al. (2020) have expressed that the nutritional value of poultry meat is improved, and growth performance is improved with additional dietary vitamin E in chickens' diets. This is especially relevant regarding the constituents and lifespan of fatty acids. Łukaszewicz et al. (2007) evaluated the impression of vitamin E and organic selenium meal administration on the sensory characteristics and chemical composition of Japanese quail breeder (*Coturnix japonica*) eggs, and they stated that the lipid content in eggs and the cholesterol concentration in yolks are reduced as a result of the addition of vitamin E and selenium into the feed of Japanese quails, while the levels of selenium, protein, and antioxidant properties are increased. Additionally, the administration of 60 IU kg^−1^ of vitamin E improves feed consumption rate; egg production percentage; and egg qualities, such as vitelline membrane strength, egg weight, and Haugh unit (Kirunda et al., 2001). A remarkable correlation exists between Se and vitamin E (VE) in terms of their protective effects on cells against free radical damage (Surai, 2002a). Additionally, the absorption of vitamin E in birds is impeded by selenium deprivation, which indicates the intricate relationship between their metabolisms (Skřivan et al., 2008). Vitamin E percentages in the plasma, egg yolk, and poultry meat are also increased by dietary selenium (Surai, 2002b). We hypothesized that different dietary sources of vegetable oils would differentially influence the productivity and egg yolk lipid profile of quail breeders and that supplementation of vitamin E would modify or improve these responses depending on the kind of oil used. This experiment was performed to determine the probable impact of sunflower oil (SUNO), linseed oil (LINO), and canola oil (CANO) with or without vitamin E treatments on the productivity parameters and egg yolk lipid metabolism of quail eggs.

## 2 Methods and materials

### 2.1 Design of an experiment

A total of 192 female Japanese quails, 10 weeks of age, were randomly allocated to six dietary treatments, with 32 quails per treatment. Each treatment was further divided into four replicates of eight hens each. This study employed a completely randomized design as a 3 × 2 factorial trial, incorporating three types of vegetable oils (SUNO 3 %, LINO 3 %, and CANO 3 %) and two doses of vitamin E (0 and 250 mg kg^−1^ diet). The fatty acid composition of the tested oils (SUNO, LINO, and CANO) according to USDA (2005) and Tarrago-Trani et al. (2006) is illustrated in Table 1.

**Table 1:** Fatty acid composition of the vegetable oil sources (SUNO, LINO, and CANO)^*^.

Fatty acid / lipid fraction	SUNO (%)	LINO (%)	CANO (%)
Palmitic acid (C16:0)	5–7	5–7	3–4
Stearic acid (C18:0)	3–5	4–6	1–2
Oleic acid (C18:1 n-9)	20–30	18–22	60–64
Linoleic acid (C18:2 n-6)	60–70	14–17	18–22
α -Linolenic acid (C18:3 n-3)	< 1	50–55	9–10
Total SFA	∼ 10–13	∼ 9–10	∼ 6–7
Total MUFA	∼ 20–30	∼ 18–22	∼ 60–64
Total PUFA	∼ 65–70	∼ 65–70	∼ 28–32
Total n-6 PUFA	∼ 60–65	∼ 13–17	∼ 18–22
Total n-3 PUFA	< 1	∼ 50–55	∼ 9–10
n-6 : n-3 ratio	Very high	Very low	Intermediate

^*^ Sources: USDA-related compositional data and published fatty acid profiles (2005) and Tarrago-Trani et al. (2006).

### 2.2 Management and experimental animals

Throughout the experimental period, quails were confined in wire-laying cages, with two hens per cage, in an open-house system. During the 10-week trial period, which covered from the age of 10 weeks to 20 weeks, all quails were maintained under identical hygienic and environmental conditions. Following a 2-week pretest phase, quail breeders were selected depending on their egg production, which surpassed 60 %. The quails were provided with a corn–soybean basal diet of 20.02 % pure protein and 2900 kcal ME kg^−1^. The formulation and analyzed nutritional composition of the basal diet, formulated according to NRC (1994), are illustrated in Table 2. The trial diets were provided to the birds in pelleted form.

**Table 2:** Composition and calculated analysis of the basal experimental diet.

Ingredients	%
Yellow corn	53.810
Soybean meal (48 %)	31.170
Di-calcium phosphate	1.161
Limestone	7.000
L-lysine HCl	0.020
Wheat bran	3.100
Sources of different vegetables oils^1^	3.000
Vit. and min. mix.^2^	0.300
Salt (NaCl)	0.300
DL- Methionine	0.139
Total	100
Calculated analyses^3^	
Crude protein, %	20.02
ME (kcal kg^−1^ diet)	2900
Ether extract, %	2.45
Crude fiber, %	2.62
Methionine, %	0.45
Methionine + cysteine, %	0.75
Lysine, %	1.02
Calcium, %	3.02
Av. Phosphorus	0.34

^1^ Sours of different vegetables oils (sunflower oil 3 %, linseed oil 3 %, and canola oil 3 %). ^2^ Each kg of the vitamin and mineral mixture contained the following: vit. A, 4 000 000 IU; vit. D_3_, 500 000 IU; vit, E, 16.7 g; vit. K, 0.67 g; vit. B1, 0.67 g; vit. B2, 2 g; vit. B6, 0.67 g; vit. B12, 0.004 g; nicotinic acid, 16.7 g; pantothenic acid, 6.67 g; biotin, 0.07 g; folic acid, 1.67 g; choline chloride, 400 g; Zn, 23.3 g; Mn, 10 g; Fe, 25 g; Cu, 1.67 g; I, 0.25 g; Se, 0.033 g; and, Mg, 133.4 g. ^3^ According to NRC (1994).

### 2.3 Measurements

#### 2.3.1 The average weight of an egg

Each day, the average was determined by weighing the eggs individually to the nearest 0.01 g for each replicate.

#### 2.3.2 Number of eggs per hen

The number of eggs laid per hen was calculated by dividing the number of chickens by the number of eggs laid for each replicate.

#### 2.3.3 Laying rate (percent)

1 $$\text{Hen-day-laying rate (HD)}\;\%=\frac{\text{Egg number}}{\text{Period (day)}}\times 100$$

The average egg weight was multiplied by the egg number to determine the egg mass.

### 2.4 Egg yolk chemical analysis

#### 2.4.1 Concentrations of total cholesterol and total lipids in egg yolks

The total lipids and yolk cholesterol were assessed at 15 and 20 weeks of age, as well as after 15 and 30 d of storage in the refrigerator. The procedure of Folch et al. (1957) was followed to extract total lipids from 0.8 g of yolk using a chloroform : methanol of 2:1 vol. / vol.. Subsequently, the concentration of cholesterol was determined using a commercial cholesterol liquid enzymatic colorimetric assay (ref. 17644H; Sentinel CH. SpA, Via Robert Koch 2, 20152 Milano, Italy) based on the Trinder method according to the manufacturer's instructions and the methodology described by Allain et al. (1974).

#### 2.4.2 Concentrations of total malondialdehyde and total antioxidant capacity in egg yolk

To detect the degree of lipid oxidation, egg yolks were examined for rhiobarbituric acid reactive substance (TBARS) concentration at the 5 and 10-week marks of the trial period. Six eggs were gathered for each treatment and saved in paper pulp egg containers at a refrigerator temperature of 4 ± 2 °C for 15 and 30 d. The 2 g of egg yolk samples were homogenized using a perchloric acid concentration of 3.86 %. A total of 50 µ L of butylated hydroxytoluene (BHT) in 4.5 % ethanol was supplied to each sample at homogenization in order to control the lipid oxidation process. After filtering the homogenate, the filtrate was mixed with distilled water and a 20 mM thiobarbituric acid reagent. Following that, the mixture was incubated. The absorbance at 531 nm was measured. Malondialdehyde milligrams per gram of yolk were used to quantify thiobarbituric acid reactive compounds (TBARSs) (Cherian et al., 2007).

### 2.5 Statistical analysis

The SAS Institute's (2005) general linear model procedures were employed to conduct analysis of variance procedures that were suitable for a wholly randomized design. The Duncan multiple-range test (Duncan, 1955) was employed to compare the means. Significant variations were defined as P < 0.05.

The subsequent model was implemented to analyze the data:

2 $$Y_{ijk}=\mu +O_{i}+A_{j}+(O\;\times \;A{)}_{ij}+E_{ijk},$$

where $Y_{ijk}$ denotes any observation, μ denotes the average, $O_{i}$ denotes the impact because of oil sources, $A_{j}$ denotes the influence of additives, ( O × A )_*i* *j*_ denotes the first-order interaction between oil sources and additives, and $E_{ijk}$ denotes the trial random error.

## 3 Results

### 3.1 Productive performance

#### 3.1.1 Egg-laying rate and egg number

Table 3 illustrates the primary impact of different sources of dietary vegetable oil, varying vitamin E treatments, and their relationship with egg production. The results demonstrated that there was no significant impact on the egg-laying rate during the trial period. Despite the incorporation of a variety of dietary vegetable oil sources, the outcomes indicated that vitamin E supplementation did not lead to any statistically significant changes ( P > 0.05) in the egg-laying rate of Japanese quail through the entire trial period. The egg-laying rate was not substantially changed by the combination of various vitamin E supplements and vegetable oil sources. The group that received canola oil achieved the highest egg production rate (71.81 %) at 15–20 weeks.

**Table 3:** Effect of various dietary vegetable oil sources, vitamin E supplementations, and their interaction on the egg production (EP, %) of laying Japanese quail hens from 10 to 20 weeks of age.

	EP % 10–15	EP % 15–20	EP % 10–20
Oil source main effect^*^			
SUNO	65.85 ± 5.027	69.56 ± 4.249	67.70 ± 3.186
LINO	68.14 ± 6.574	69.83 ± 9.899	68.99 ± 7.815
CANO	68.35 ± 4.312	70.83 ± 10.23	69.59 ± 5.353
P value	0.626	0.959	0.827
Antioxidant main effect			
without VE	66.82 ± 5.860	70.90 ± 8.367	68.86 ± 6.523
VE	68.07 ± 4.798	69.25 ± 8.343	68.66 ± 4.726
P value	0.593	0.665	0.939
Interaction effect			
SUNO without VE	63.79 ± 4.629	70.94 ± 5.977	67.36 ± 4.075
SUNO + VE	67.91 ± 5.121	68.18 ± 1.145	68.04 ± 2.602
LINO without VE	68.21 ± 8.580	69.95 ± 11.22	69.08 ± 9.555
LINO + VE	68.07 ± 5.216	69.72 ± 10.14	68.89 ± 7.153
CANO without VE	68.45 ± 3.531	71.81 ± 9.633	70.13 ± 6.549
CANO + VE	68.24 ± 5.558	69.85 ± 12.20	69.05 ± 4.814
P value	0.686	0.962	0.961

Means in the same column with no superscript letters after them or with a common superscript letter following them are not significantly different ( P < 0.05). ^*^ SUNO: sunflower oil, 3 %; LINO: flaxseed oil, 3 %; CANO: canola oil, 3 %.

The egg number (egg per hen per day) of Japanese quail hens was influenced by a variety of sources of dietary vegetable oil, varying vitamin E treatments, and their combination, as demonstrated in Table 4. The egg number (eggs per hen per day) of quail hens did not change ( P > 0.05) as a result of the incorporation of a variety of dietary vegetable oil sources into the feed throughout the entirety of the trial. The results indicated that the egg weight generated by laying hens in various treatment groups was affected by varying vitamin E supplementations, irrespective of the source of dietary vegetable oil. The data indicated that there was no interaction effect between several dietary vegetable oil sources and distinct antioxidant supplements in terms of egg number ( P > 0.05). Nevertheless, the laying hens that were administered either 3 % canola oil alone or with the supplementation of vitamin E into the feed produced the highest numerical egg count (0.718 and 0.698 eggs per hen per day) during the majority of the experimental periods, as indicated by general observation.

**Table 4:** Effect of various dietary vegetable oil sources, vitamin E supplementation, and their interaction on egg number (EN, eggs per hen per day) of laying Japanese quail hens during the period from 10 to 20 weeks of age.

	EN 10–15	EN 15–20	EN 10–20
Oil source main effect^*^			
SUNO	0.658 ± 0.050	0.696 ± 0.042	0.677 ± 0.032
LINO	0.681 ± 0.066	0.698 ± 0.099	0.690 ± 0.078
CANO	0.683 ± 0.043	0.708 ± 0.102	0.696 ± 0.054
P value	0.626	0.959	0.827
Antioxidant main effect			
without VE	0.668 ± 0.059	0.709 ± 0.084	0.689 ± 0.065
VE	0.681 ± 0.048	0.692 ± 0.083	0.687 ± 0.047
P value	0.593	0.665	0.939
Interaction effect			
SUNO without VE	0.638 ± 0.046	0.709 ± 0.060	0.674 ± 0.041
SUNO + VE	0.679 ± 0.051	0.682 ± 0.011	0.680 ± 0.026
LINO without VE	0.682 ± 0.086	0.700 ± 0.112	0.691 ± 0.096
LINO + VE	0.681 ± 0.052	0.697 ± 0.101	0.689 ± 0.072
CANO without VE	0.685 ± 0.035	0.718 ± 0.096	0.701 ± 0.065
CANO + VE	0.682 ± 0.056	0.698 ± 0.122	0.690 ± 0.048
P value	0.686	0.962	0.961

Means in the same column with no superscript letters after them or with a common superscript letter following them are not significantly different ( P < 0.05). ^*^ SUNO: sunflower oil, 3 %; LINO: flaxseed oil, 3 %; CANO: canola oil, 3 %.

**Table 5:** Effect of various dietary vegetable oil sources, vitamin E supplementation, and their interaction on egg weight (EW, g) of laying Japanese quail hens from 10 to 20 weeks of age.

	EW 10–15	EW 15–20	EW 10–20
Oil source main effect^*^			
SUNO	9.932 ± 1.396	10.27 ± 0.681^b^	10.09 ± 0.833^b^
LINO	10.88 ± 0.969	12.10 ± 1.907^a^	11.51 ± 0.978^a^
CANO	9.571 ± 0.901	12.20 ± 0.971^a^	10.92 ± 0.715^a^
P value	0.063	0.002	0.007
Antioxidant main effect			
without VE	10.55 ± 1.099	11.22 ± 1.344	10.88 ± 0.843
VE	9.709 ± 1.196	11.83 ± 1.710	10.80 ± 1.180
P value	0.070	0.166	0.807
Interaction effect			
SUNO without VE	10.05 ± 1.404	10.13 ± 0.815^d^	10.08 ± 0.737
SUNO + VE	9.815 ± 1.593	10.41 ± 0.608^cd^	10.11 ± 1.038
LINO without VE	11.35 ± 1.035	10.80 ± 0.571^cd^	11.06 ± 0.743
LINO + VE	10.41 ± 0.727	13.41 ± 1.900^a^	11.95 ± 1.074
CANO without VE	10.24 ± 0.249	12.73 ± 0.891^ab^	11.50 ± 0.341
CANO + VE	8.900 ± 0.796	11.67 ± 0.816^bc^	10.35 ± 0.445
P value	0.584	0.008	0.054

Means in the same column with no superscript letters after them or with a common superscript letter following them are not significantly different ( P < 0.05). ^*^ SUNO: sunflower oil, 3 %; LINO: flaxseed oil, 3 %; CANO: canola oil, 3 %.

#### 3.1.2 The average weight of an egg

The egg weight (g) of Japanese quail breeders was affected by different sources of vegetable oil, variable levels of vitamin E supplementation, and their interaction, as illustrated in Table 5. The egg weight generated by laying hens across different vegetable-oil-inclusive diet groups was substantially different ( P < 0.05) at 15–20 and 10–20 weeks. Dietary vegetable oil sources and various supplements of vitamin E exhibit a significant interaction ( P < 0.05) that influences egg weight throughout the time of the trial. The hens that were administered 3 % linseed and vitamin E or 3 % canola oil without vitamin E gave increased egg weight (13.41 and 12.73g) at 15–20 weeks.

#### 3.1.3 Egg mass

Table 6 illustrates the influences of different sources of dietary vegetable oil, variable doses of vitamin E treatments, and their combination on the egg mass of Japanese quail. The use of several dietary vegetable oil sources did not substantially ( P > 0.05) influence the average daily egg mass of chickens, and the group supplemented with canola oil and linseed oil exhibited high results (8.657 and 8.541 g, respectively) at 15–20 weeks. The results indicated that the egg mass (g per hen per day) generated by laying hens in various treatment groups was insignificantly affected ( P > 0.05) by varying vitamin E supplementations, irrespective of the source of dietary vegetable oil. The birds that were fed a diet that contained vitamin E exhibited the highest egg mass (8.272 g) at 15–20 weeks. Furthermore, dietary vegetable oil sources and various supplements did not interact substantially ( P > 0.05), and the groups treated with linseed oil with vit. E and canola oil without vit. E exhibited high results (9.493 and 9.086 g and 8.282 and 8.049 g, respectively) during the periods of 15–20 and 10–20 weeks of age.

**Table 6:** Effect of various dietary vegetable oil sources, vitamin E supplementation, and their interaction on egg mass (EM, g) of laying Japanese quail hens from 10 to 20 weeks of age.

	EM 10–15	EM 15–20	EM 10–20
Oil source main effect^*^			
SUNO	6.526 ± 0.922	7.133 ± 0.500	6.829 ± 0.572
LINO	7.435 ± 1.201	8.541 ± 2.271	7.988 ± 1.490
CANO	6.544 ± 0.745	8.657 ± 1.517	7.600 ± 0.741
P value	0.129	0.130	0.103
Antioxidant main effect			
without VE	7.063 ± 1.145	7.947 ± 1.287	7.505 ± 1.058
VE	6.607 ± 0.884	8.272 ± 2.059	7.440 ± 1.164
P value	0.265	0.621	0.879
Interaction effect			
SUNO without VE	6.378 ± 0.712	7.168 ± 0.574	6.773 ± 0.223
SUNO + VE	6.673 ± 1.192	7.097 ± 0.501	6.885 ± 0.839
LINO without VE	7.799 ± 1.667	7.588 ± 1.568	7.693 ± 1.615
LINO + VE	7.071 ± 0.486	9.493 ± 2.675	8.282 ± 1.530
CANO without VE	7.011 ± 0.417	9.086 ± 0.757	8.049 ± 0.538
CANO + VE	6.076 ± 0.734	8.227 ± 2.074	7.151 ± 0.674
P value	0.417	0.226	0.363

Means in the same column with no superscript letters after them or with a common superscript letter following them are not significantly different ( P < 0.05). ^*^ SUNO: sunflower oil, 3 %; LINO: flaxseed oil, 3 %; CANO: canola oil, 3 %.

#### 3.1.4 Feed intake and feed conversion ratio (FCR)

Table 7 illustrates the impact of the interaction between vitamin E supplementation, various dietary vegetable oil sources, and feed intake (FI) on Japanese quail breeders from the age of 10 to 20 weeks. The results indicate that there is a substantial difference ( P < 0.05) in feed intake due to the supplementation of various oil sources. The group that received sunflower oil showed an increase in feed intake (28.97 g), while the group that received linseed oil exhibited a decline in feed intake (27.93 g) throughout the experimental period at 10–20 weeks of age. Conversely, vitamin E supplementation demonstrated a non-significant alteration ( P > 0.05) in feed intake throughout the trial period. Additionally, the interaction between dietary vegetable oil sources and various vitamin E supplements developed a non-significant change ( P > 0.05) in feed intake throughout the trial.

**Table 7:** Effect of various dietary vegetable oil sources, vitamin E supplementation, and their interaction on feed intake (FI, g) of laying Japanese quail hens from 10 to 20 weeks of age.

	FI 10–15	FI 15–20	FI 10–20
Oil source main effect^*^			
SUNO	29.13 ± 0.640^a^	28.80 ± 0.636^a^	28.97 ± 0.502^a^
LINO	27.94 ± 0.421^c^	27.92 ± 0.415^b^	27.93 ± 0.215^c^
CANO	28.51 ± 0.671^b^	28.23 ± 0.403^b^	28.37 ± 0.432^b^
P value	0.001	0.009	0.000
Antioxidant main effect			
without VE	28.79 ± 0.828^a^	28.26 ± 0.602	28.52 ± 0.634
VE	28.27 ± 0.586^b^	28.38 ± 0.628	28.32 ± 0.531
P value	0.023	0.582	0.217
Interaction effect			
SUNO without VE	29.53 ± 0.259	28.73 ± 0.617	29.13 ± 0.388
SUNO + VE	28.74 ± 0.690	28.88 ± 0.739	28.81 ± 0.610
LINO without VE	27.94 ± 0.619	27.72 ± 0.210	27.83 ± 0.226
LINO + VE	27.95 ± 0.177	28.12 ± 0.505	28.03 ± 0.172
CANO without VE	28.90 ± 0.589	28.33 ± 0.474	28.61 ± 0.362
CANO + VE	28.11 ± 0.536	28.13 ± 0.358	28.12 ± 0.380
P value	0.227	0.524	0.199

Means in the same column with no superscript letters after them or with a common superscript letter following them are not significantly different ( P < 0.05). ^*^ SUNO: sunflower oil, 3 %; LINO: flaxseed oil, 3 %; CANO: canola oil, 3 %.

The FCR of Japanese quail breeders from the age of 10 to 20 weeks is illustrated in Table 8, which also illustrates the impact of vitamin E supplementation, various sources from dietary vegetable oil, and their combination. The outcomes demonstrated a substantial boost ( P=0.038 ) in the feed conversion ratio because of the supplementation of various oil sources in the diet. The group that received linseed oil exhibited the best results (3.6), while the group that received sunflower oil exhibited the worst results (4.271) from the age of 10 to 20 weeks. Additionally, the FCR demonstrated a non-significant increase ( P=0.904 ) from 10 to 20 weeks of age because of the vitamin E supplementation. Moreover, the interaction between different dietary sources from vegetable oil and different vitamin E treatments exposed a non-significant progress ( P=0.426 ) in FCR throughout the trial period. The group that received linseed oil with vitamin E exhibited the best result (3.474), while the group that received sunflower oil without vitamin E supplementation exhibited the worst results (4.304) from the age of 10 to 20 weeks.

**Table 8:** Effect of various dietary vegetable oil sources, vitamin E supplementation, and their interaction on feed conversion ratio (FCR, g feed per g egg) of laying Japanese quail hens from 10 to 20 weeks of age.

	FCR 10–15	FCR 15–20	FCR 10–20
Oil source main effect^*^			
SUNO	4.548 ± 0.675	4.056 ± 0.311	4.271 ± 0.408^a^
LINO	3.830 ± 0.523	3.454 ± 0.812	3.600 ± 0.643^b^
CANO	4.409 ± 0.544	3.347 ± 0.570	3.762 ± 0.348^ab^
P value	0.060	0.061	0.038
Antioxidant main effect			
without VE	4.165 ± 0.626	3.639 ± 0.572	3.865 ± 0.516
VE	4.359 ± 0.672	3.599 ± 0.757	3.890 ± 0.598
P value	0.431	0.870	0.904
Interaction effect			
SUNO without VE	4.669 ± 0.479	4.025 ± 0.301	4.304 ± 0.184
SUNO + VE	4.426 ± 0.891	4.088 ± 0.364	4.238 ± 0.593
LINO without VE	3.693 ± 0.712	3.762 ± 0.703	3.727 ± 0.708
LINO + VE	3.967 ± 0.286	3.146 ± 0.890	3.474 ± 0.649
CANO without VE	4.133 ± 0.248	3.131 ± 0.223	3.565 ± 0.198
CANO + VE	4.685 ± 0.653	3.563 ± 0.764	3.958 ± 0.374
P value	0.413	0.233	0.426

Means in the same column with no superscript letters after them or with a common superscript letter following them are not significantly different ( P < 0.05). ^*^ SUNO: sunflower oil, 3 %; LINO: flaxseed oil, 3 %; CANO: canola oil, 3 %.

### 3.2 The total lipid content of egg yolks

Table 9 delineates the impact of various sources of vegetable oil, vitamin E administration, and their interaction on the total lipid content of egg yolks at the age of 15 and 20 weeks, as well as following 15 and 30 d of refrigeration. The total lipids in egg yolks were diminished at both 15 and 20 weeks of age and after 15 and 30 d of refrigeration because of the integration of various vegetable oil sources in the feed. The group receiving canola oil at 15 and 20 weeks of age and after 15 and 30 d of refrigeration demonstrated minimal ( P < 0.0001) total lipid values in egg yolks. This reduction was 209.2, 248.2, 220, and 210.7, respectively. Different vitamin E supplementations substantially reduced the total lipids of egg yolks at the ages of 15 and 20 weeks and after 15 d of storage in the refrigerator, whatever the source of vegetable oil ( P=0.051 ). The total lipid content of egg yolks is substantially influenced by the interaction between dietary vegetable oil sources and various vitamin E supplements ( P < 0.05). The breeders that were fed diets with 3 % canola oil and 250 mg vitamin E kg^−1^ diet demonstrated minimal egg yolk total lipid content at the ages of 15 and 20 weeks, as well as after 15 d of storage in the refrigerator, and the groups supplemented with linseed oil with vitamin E demonstrated decreased levels of egg yolk total lipids (216.7) after 15 d of storage in the refrigerator, while the groups supplemented with canola oil with vitamin E demonstrated decreased levels of egg yolk total lipids (210) after 30 d of storage in the refrigerator.

**Table 9:** Effect of various dietary vegetable oil sources, vitamin E supplementation, and their interaction on yolk total lipid of Japanese quail laying hens at 15 and 20 weeks of age and after 15 and 30 d storage in the refrigerator.

	Total lipids 15 w	Total lipids 20 w	Total lipids 15 d	Total lipids 30 d
Oil source main effect^*^				
SUNO	344.0 ± 20.04^a^	318.2 ± 15.98^a^	258.0 ± 32.66^a^	275.5 ± 15.24^a^
LINO	306.5 ± 5.541^b^	254.0 ± 37.52^b^	260.2 ± 48.74^a^	225.2 ± 10.46^b^
CANO	209.2 ± 8.519^c^	248.2 ± 12.01^b^	220.0 ± 8.222^b^	210.7 ± 14.05^b^
P value	0.000	0.001	0.001	0.000
Antioxidant main effect				
without VE	291.3 ± 61.09	281.2 ± 43.44	253.6 ± 40.99	240.8 ± 36.38
VE	281.8 ± 61.62	265.7 ± 37.01	238.6 ± 33.94	233.4 ± 26.83
P value	0.109	0.216	0.051	0.226
Interaction effect				
SUNO without VE	354.0 ± 11.53	331.7 ± 3.055	233.7 ± 24.50^b^	287.7 ± 11.06
SUNO + VE	334.0 ± 23.90	304.7 ± 9.074	282.3 ± 17.01^a^	263.3 ± 3.786
LINO without VE	304.3 ± 7.095	259.7 ± 38.50	303.7 ± 16.04^a^	223.3 ± 10.60
LINO + VE	308.7 ± 3.512	248.3 ± 44.05	216.7 ± 2.082^b^	227.0 ± 12.29
CANO without VE	215.7 ± 3.512	252.3 ± 17.01	223.3 ± 11.55^b^	211.3 ± 2.309
CANO + VE	202.7 ± 6.506	244.0 ± 4.359	216.7 ± 1.528^b^	210.0 ± 22.07
P value	0.220	0.793	0.000	0.148

Means in the same column with no superscript letters after them or with a common superscript letter following them are not significantly different ( P < 0.05). ^*^ SUNO: sunflower oil, 3 %; LINO: flaxseed oil, 3 %; CANO: canola oil, 3 %.

#### 3.2.1 The total cholesterol content of egg yolk

Table 10 demonstrates the impact of interventions on the total cholesterol percentage in egg yolks at the ages of 15 and 20 weeks, as well as during 15 and 30 d of refrigeration. The experimental findings at 15 weeks of age demonstrated a beneficial impact of different vegetable oil sources, significantly decreasing the total cholesterol concentrations in the egg yolk ( P=0.004 ). The group that was supplemented with canola oil exhibited decreased levels. In contrast to the other group, this beneficial effect was lost at 20 weeks of age and after 15 and 30 d of refrigerator storage. Different vitamin E supplementations at 15 weeks of age reduced the total lipid content of the egg yolk, irrespective of the source of vegetable oil. Nevertheless, this beneficial effect was absent ( P > 0.05) at the age of 20 weeks and after 15 and 30 d of refrigerator storage in comparison to the other group. The cholesterol concentrations of egg yolks at the age of 15 weeks were substantially reduced ( P=0.004 ) by various antioxidant supplements and vegetable oil sources, as indicated by the results, and the group supplemented with canola oil revealed the decreased results (16). In contrast to the other group, this positive result was absent ( P > 0.05) at the age of 20 weeks and after 15 and 30 d of storage in the refrigerator, and the group supplemented with canola oil supplemented with vitamin E revealed decreased results (14.67) at 20 weeks of age.

**Table 10:** Effect of various dietary vegetable oil sources, vitamin E supplementation, and their interaction on yolk cholesterol of Japanese quail laying hens at 15 and 20 weeks of age and after 15 and 30 d storage in the refrigerator.

	Cholesterol 15 w	Cholesterol 20 w	Cholesterol 15 d	Cholesterol 30 d
Oil source main effect^*^				
SUNO	17.83 ± 0.753^b^	17.00 ± 2.828	17.67 ± 1.033	17.67 ± 2.733
LINO	21.50 ± 4.848^a^	17.50 ± 2.074	16.67 ± 2.503	18.33 ± 1.966
CANO	16.00 ± 3.225^b^	15.17 ± 1.941	17.00 ± 1.897	17.00 ± 3.098
P value	0.004	0.196	0.671	0.638
Antioxidant main effect				
without VE	17.44 ± 2.128	17.00 ± 2.000	17.78 ± 2.108	18.33 ± 2.179
VE	19.44 ± 5.151	16.11 ± 2.804	16.44 ± 1.333	17.00 ± 2.828
P value	0.087	0.408	0.171	0.260
Interaction effect				
SUNO without VE	18.00 ± 1.000^b^	18.67 ± 1.528	18.33 ± 0.577	16.67 ± 1.155
SUNO + VE	17.67 ± 0.577^b^	15.33 ± 3.055	17.00 ± 1.000	18.67 ± 3.786
LINO without VE	17.33 ± 0.577^b^	16.67 ± 1.528	17.00 ± 3.606	19.33 ± 1.528
LINO + VE	25.67 ± 2.517^a^	18.33 ± 2.517	16.33 ± 1.528	17.33 ± 2.082
CANO without VE	17.00 ± 4.000^b^	15.67 ± 2.082	18.00 ± 1.732	19.00 ± 3.000
CANO + VE	15.00 ± 2.646^b^	14.67 ± 2.082	16.00 ± 1.732	15.00 ± 1.732
P value	0.004	0.186	0.840	0.128

Means in the same column with no superscript letters after them or with a common superscript letter following them are not significantly different ( P < 0.05). ^*^ SUNO: sunflower oil, 3 %; LINO: flaxseed oil, 3 %; CANO: canola oil, 3 %.

#### 3.2.2 Concentration of malondialdehyde in egg yolk

Table 11 summarizes the effects of several vegetable oil sources on the malondialdehyde percentage (nmol g^−1^ yolk) in the yolk of Japanese quails at 15, 20, and 30 weeks of age, as well as after 15 and 30 d of refrigeration storage. The concentration of malondialdehyde in egg yolks decreased at 15 and 20 weeks of age, as well as at 15 and 30 d post-refrigeration, due to the addition of different vegetable oil sources into the diet. The group that was treated with canola oil at 15 weeks of age and linseed oil at 30 d after storage in the refrigerator recorded a minimal concentration of egg yolk malondialdehyde (3.775 and 3.950 nmol g^−1^ yolk; P=0.031 , P=0.007 ). The oxidative stability of the fresh Japanese quail yolk was determined to be progressively worse as the storage period was extended. Additionally, the yolk's susceptibility to oxidation was identified when vitamin E was supplied to the diet. In the majority of the samplings, the malondialdehyde values of the quails that were fed diets supplemented with vitamin E were substantially lower ( P < 0.05) than those of other groups.

**Table 11:** Effect of various dietary vegetable oil sources, vitamin E supplementation, and their interaction on yolk malondialdehyde (MDA) of Japanese quail laying hens at 15 and 20 weeks of age and after 15 and 30 d storage in the refrigerator.

	MDA 15 w	MDA 20 w	MDA 15 d	MDA 30 d
Oil source main effect^*^				
SUNO	4.025 ± 0.366	4.000 ± 0.420^ab^	3.900 ± 0.341	4.250 ± 0.214^a^
LINO	4.075 ± 0.268	4.250 ± 0.207^a^	3.925 ± 0.133	3.850 ± 0.217^b^
CANO	3.875 ± 0.300	3.775 ± 0.256^b^	3.700 ± 0.406	3.950 ± 0.272^b^
P value	0.420	0.031	0.162	0.007
Antioxidant main effect				
without VE	3.867 ± 0.325	3.933 ± 0.361	3.667 ± 0.338^b^	4.000 ± 0.273
VE	4.117 ± 0.245	4.083 ± 0.346	4.017 ± 0.162^a^	4.033 ± 0.307
P value	0.067	0.259	0.004	0.705
Interaction effect				
SUNO without VE	3.800 ± 0.400	3.700 ± 0.400	3.700 ± 0.400^bc^	4.150 ± 0.150^ab^
SUNO + VE	4.250 ± 0.150	4.300 ± 0.100	4.100 ± 0.100^a^	4.350 ± 0.250^a^
LINO without VE	4.150 ± 0.050	4.300 ± 0.100	3.950 ± 0.050^ab^	3.700 ± 0.100^c^
LINO + VE	4.000 ± 0.400	4.200 ± 0.300	3.900 ± 0.200^ab^	4.000 ± 0.200^bc^
CANO without VE	3.650 ± 0.250	3.800 ± 0.200	3.350 ± 0.150^c^	4.150 ± 0.250^ab^
CANO + VE	4.100 ± 0.100	3.750 ± 0.350	4.050 ± 0.150^ab^	3.750 ± 0.050^c^
P value	0.116	0.078	0.027	0.013

Means in the same column with no superscript letters after them or with a common superscript letter following them are not significantly different ( P < 0.05). ^*^ SUNO: sunflower oil, 3 %; LINO: flaxseed oil, 3 %; CANO: canola oil, 3 %.

#### 3.2.3 The concentration of total antioxidant capacity in egg yolks

Table 12 displays that the egg yolk concentration of total antioxidant capacity was raised at the ages of 15 and 20 weeks, as well as at 15 and 30 d after storage in the refrigerator, as a result of the addition of various vegetable oil sources into the diet. In comparison to the other group, the group that was treated with linseed oil at 20 weeks of age and the group that received canola oil at 30 d after storage in a refrigerator exhibited the maximum ( P < 0.01) egg yolk total antioxidant capacity concentration. The total antioxidant capacity concentration of egg yolks was significantly influenced by various antioxidant supplements at 15 weeks of age and after 15 and 30 d of refrigerator storage when the impact of vegetable oil sources was disregarded. The groups that received vitamin E after 15 d of refrigerator storage achieved the increased (406 nmol g^−1^ yolk) value of egg yolk total antioxidant capacity concentration ( P=0.043 ). The interaction effect of the several treatments that were examined on the percentage of egg yolks' total antioxidant capacity was found to be P < 0.005. The group that was treated with linseed oil and vitamin E exhibited the highest antioxidant capacity at 15, 20, and 15 d of refrigerator storage (0.409, 0.409, and 0.402 nmol g^−1^ yolk). The groups that were fed linseed or canola and treated with vitamin E recorded the maximum levels at 15 and 20 weeks of age, as well as after 15 and 30 d of storage in the refrigerator.

**Table 12:** Effect of various dietary vegetable oil sources, different antioxidant supplementations, and their interaction on yolk total antioxidant capacity of Japanese quail laying hens at 15 and 20 weeks of age and after 15 and 30 d storage in the refrigerator.

	TAC 15 w	TAC 20 w	TAC 15 d	TAC 30 d
Oil source main effect^*^				
SUNO	0.395 ± 0.008	0.407 ± 0.010^a^	409.5 ± 5.320^a^	0.403 ± 0.012^b^
LINO	0.400 ± 0.011	0.402 ± 0.008^a^	392.0 ± 12.66^b^	0.388 ± 0.006^c^
CANO	0.403 ± 0.009	0.390 ± 0.003^b^	405.5 ± 8.167^a^	0.410 ± 0.002^a^
P value	0.100	0.000	0.002	0.000
Antioxidant main effect				
without VE	0.397 ± 0.010	0.399 ± 0.012	398.7 ± 12.83^b^	0.405 ± 0.010^a^
VE	0.402 ± 0.008	0.400 ± 0.010	406.0 ± 9.539^a^	0.395 ± 0.012^b^
P value	0.158	0.890	0.043	0.000
Interaction effect				
SUNO without VE	0.391 ± 0.004^c^	0.414 ± 0.003^a^	406.0 ± 5.000^a^	0.414 ± 0.003^a^
SUNO + VE	0.400 ± 0.010^bc^	0.400 ± 0.010^b^	413.0 ± 3.000^a^	0.392 ± 0.001^c^
LINO without VE	0.391 ± 0.003^c^	0.395 ± 0.004^bc^	382.0 ± 1.000^b^	0.393 ± 0.002^c^
LINO + VE	0.409 ± 0.007^ab^	0.409 ± 0.001^a^	402.0 ± 10.00^a^	0.383 ± 0.002^d^
CANO without VE	0.411 ± 0.004^a^	0.389 ± 0.003^c^	408.0 ± 2.000^a^	0.410 ± 0.003^b^
CANO + VE	0.396 ± 0.003^c^	0.390 ± 0.004^c^	403.0 ± 12.00^a^	0.410 ± 0.001^b^
P value	0.001	0.002	0.027	0.000

Means in the same column with no superscript letters after them or with a common superscript letter following them are not significantly different ( P < 0.05). ^*^ SUNO: sunflower oil, 3 %; LINO: flaxseed oil, 3 %; CANO: canola oil, 3 %.

## 4 Discussion

The nearly identical egg production in Japanese quail breeders with various dietary supplementations may be attributed to the fact that all feed was maintained to have the same percentage of energy and protein, as well as being similar with regard to other essential nutrients (Ebeid et al., 2011). Vegetable oils including SUNO, LINO, and CANO can affect the productivity of laying hens, primarily through their fatty acid profiles; SUNO, rich in linoleic acid, supports yolk n-6 PUFA deposition and energy supply; LINO, rich in α -linolenic acid, improves yolk n-3 PUFA and enhances the ratio of n-6 : n-3; and CANO provides moderate PUFA and oleic acid. These oils may also modulate antioxidant defenses and lipid metabolism (Mazalli et al., 2004; Saleh et al., 2013).

These outcomes align with the outputs of Silke et al. (2008), who asserted that the egg production of hens that were fed either 10 % or 20 % flaxseed in their diets was not substantially different ( P > 0.05). Additionally, according to the findings of Jalil and Yurtseven (2023), no considerable variation in egg production was observed across the different concentrations ( P > 0.05). The highest average total number of eggs laid over 14 d occurred with the application of moringa leaf, whereas the lowest was observed with canola seed powder. In contrast, Celebi and Utlu (2006) evidenced a significant enhancement ( P < 0.05) in egg production among chickens supplemented with linseed oil. However, another study showed a decreasing in egg production in chickens that were fed diets supplemented with linseed meal or linseed oil (Švedová et al., 2008).

These discoveries align with those of Golzar Adabi et al. (2016), who determined that the egg weight and laying rate were not substantially impacted by canola and linseed oils ( P > 0.05). Zeweil et al. (2013) discovered that the addition of linseed meal into the feed did not produce a statistically substantial variation in the egg-laying rate, egg weight, and egg mass of laying Japanese quails when contrasted with laying hens that were fed a diet devoid of flaxseed meal. Throughout the egg-laying phase of the Japanese quail, natural supplements, including linseed oil, canola oil, and vitamin E, had a beneficial impact on the antioxidative qualities of blood and egg yolks, as well as efficacy and lipid metabolism, as detailed by Abo Egila et al. (2023). Vitamin E may be capable of inhibiting oxidative damage and initiating the release of the yolk precursor protein, which is crucial for the development of eggs, by maintaining the integrity of the liver cells and other organs activity (Abd-El-Maksoud, 2006). In addition, Omar (2014) discovered that the total lipids, triglycerides, and cholesterol concentrations of egg yolks in laying Japanese quails at 24 weeks of age were substantially reduced by the addition of linseed and vitamins A and E into the feed. Behesti Moghadam et al. (2019) observed that the total lipid content of eggs was not influenced by the heating of flaxseed or enzyme supplementation. The discovery that linseed oil augmented egg production in stressed chickens was intriguing. In hens, the hypothalamic–pituitary–gonadal gland regulates the egg-laying process, which is stimulated by the gonadotropin-releasing hormone (Mishra et al., 2019). With the oral addition of essential oil extracted from ginger rhizomes to Japanese quails, Tchoffo et al. (2019) discovered that yolk cholesterol was reduced. The egg yolk accumulates fatty acids from vegetable oils, which leads to lipid oxidation, hydrolysis, and polymerization (Wang et al., 2017). Consequently, sensory properties such as taste, flavor, color, and texture are impacted (Brelaz et al., 2019). Conversely, stress exacerbated lipid oxidation (Eid et al., 2008), which could potentially modify sensory characteristics. Vitamin E and selenium may exert a protective effect on lipid peroxidation due to their antioxidant properties in glutathione peroxidase activity. In the cytosol and mitochondrial matrix, the reduced form of GSH-Px contributes to the detoxification of pro-oxidants by reducing hydrogen peroxide and lipid hydroperoxide (Bansal and Kaur, 2005). Mohiti-Asli et al. (2008), in accordance with our data, observed that the elevated MDA content in eggs maintained in an incubator and at ambient temperature can be alleviated by maternal dietary administration of selenium and vitamin E.

To our knowledge, no data exist demonstrating the contrary impacts of stocking density stress on the sensory characteristics of eggs. Behesti Moghadam et al. (2019) stated that the levels of thiobarbituric acid reactive compounds in eggs produced from layers fed heated linseed combined with vitamin E (60 °C for 8 h) were substantially lower than those in eggs produced from layers fed raw flaxseed with vitamin E ( P < 0.05). Upon 10 d of storage at room temperature, Faitarone et al. (2016) noted a significant rise in yolk lipid oxidation. The eggs from layers fed on a diet without oil administration or supplied with 2.5 % soybean oil had a reduced level of yolk lipid oxidation compared to those from layers fed on diets treated with 5 % linseed oil and 2.5 % canola oil plus 2.5 % soybean oil. Nonetheless, these eggs exhibited no substantial differences. Giampietro et al. (2008) confirmed that the oxidation of lipids in egg yolks escalates with the age of the egg. The authors recorded thiobarbituric acid reactive (TBA) substance levels of 0.1343 in raw eggs, which rose to 0.1698 after 7 d of storage and to 0.2138 after 14 d of storage. Our findings are at odds with those of Gómez (2003), who observed that the lipid oxidation levels of the eggs of hens supplemented with diets with 5 % flaxseed oil were considerably higher than those of chicken fed diets with no oil administration. The researcher posits that the increased levels of oxidation of yolk lipids are due to the increased levels of unsaturated linolenic acid. Roux et al. (2011) demonstrated that omega-3 enhancement elevates MDA levels in the absence of vitamin E supplementation. Nevertheless, the MDA values remained consistent with those of the control group, which contained no omega-3 or vitamin E supplementation. This was because of the addition of vitamin E, and MDA levels increased during refrigerated storage. Moreover, the characteristics under investigation exhibited a substantial interaction effect, and the groups that were fed linseed or canola and treated with vitamin E recorded the highest levels of MDA at 15 and 20 weeks of age, as well as after 15 and 30 d of storage in the refrigerator.

The lipid oxidation constancy of fresh quail eggs is generally affected by various supplements that are included in the diet during the egg's storage in the refrigerator under the circumstances of the current trial. The existing conclusions agreed with those of Narciso-Gaytán et al. (2010) to a lesser or greater extent. They reported that the high supplemented level showed lower ( P ≤ 0.05) malonaldehyde values than the control treatment. In conclusion, the lipid oxidation stability of chicken meat is influenced by the lipid source and vitamin E level included in the diet upon storage time and processing of the meat.

Tchoffo et al. (2019) recently discovered that the concentration of yolk MDA decreased substantially ( P < 0.05) as the dose of ginger essential oil increased. Unlike MDA, the GPx activity status increased linearly and substantially ( P < 0.05) as the essential oil levels increased. The MDA is a significant endogenous lipid peroxidation product; consequently, the degree of fat peroxidation by reactive oxygen species (ROSs) may be observed in MDA concentrations (Jiang et al., 2007). Nevertheless, the concentration of antioxidants in developing embryonic tissues is increased by a diet that contains antioxidant properties, which, in turn, decreases their liability with regard to lipid peroxidation (Surai, 1999). Consequently, the rate of embryonic mortality is reduced. In the present study, ginger was found to increase the antioxidant capability of birds against ROSs and to decrease plasma MDA levels in comparison to the control group, as noted by Akbarian et al. (2011). Previously, Tchoffo et al. (2019) discovered that the GPx action was amplified linearly and substantially ( P < 0.05) as the essential oil levels increased. Relative to the control, the CAT action was enhanced by ginger essential oil at all chosen dosages. Nevertheless, this rise was statistically significant ( P < 0.05) only at 100 µ L kg^−1^ body weight. Additionally, the MDA concentrations in the egg yolk were slightly lowered by the inclusion of 800 mg vitamin E in the diets of laying hens by Tadesse et al. (2023).

## 5 Conclusion

The information gained from this research demonstrated that the combined use of flaxseed oil, canola oil, and vitamin E had positive effects on the productivity, lipid metabolism, and antioxidative properties of egg yolks in Japanese quail breeders (*Coturnix japonica*) within the laying period.

## 6 Declarations

### 6.1 Ethics approval and consent to participate

The current experimental investigation was performed at the Poultry Research Laboratory, a division of the Animal and Fish Production Department within the Faculty of Agriculture (Saba Basha) at Alexandria University, Egypt. We obtained informed consent from the owner of the birds involved in the study. All methods were carried out according to relevant guidelines and regulations (Ethics Committee, Faculty of Agriculture Alexandria University, Egypt). The study was carried out in compliance with the ARRIVE guidelines.

## Data Availability

The data presented in this study are available on request from the corresponding authors.
